# Challenges and Opportunities in Hydrometallurgical Recovery of Germanium from Coal By-Products

**DOI:** 10.3390/molecules30081695

**Published:** 2025-04-10

**Authors:** Ewa Rudnik

**Affiliations:** Faculty of Non-Ferrous Metals, AGH University of Krakow, Mickiewicz Ave. 30, 30-059 Krakow, Poland; erudnik@agh.edu.pl

**Keywords:** coal fly ash, combustion, gasification, leaching, recovery, separation

## Abstract

Germanium, a critical material for advanced technologies, is enriched in certain coal deposits and by-products, including coal combustion and gasification fly ashes. This review examines germanium concentrations and occurrence modes in coal, coal gangue, and their combustion or gasification by-products, as well as hydrometallurgical recovery methods at laboratory, pilot, and industrial scales. Fly ashes from both coal combustion and gasification are particularly promising due to their higher germanium content and recovery rates, which can exceed 90% under optimal conditions. However, the low germanium concentrations and high levels of impurities in the leachates pose challenges, necessitating the development of innovative and selective separation techniques, primarily involving solvent extraction, ion exchange, or adsorption.

## 1. Introduction

**Figure 1:**
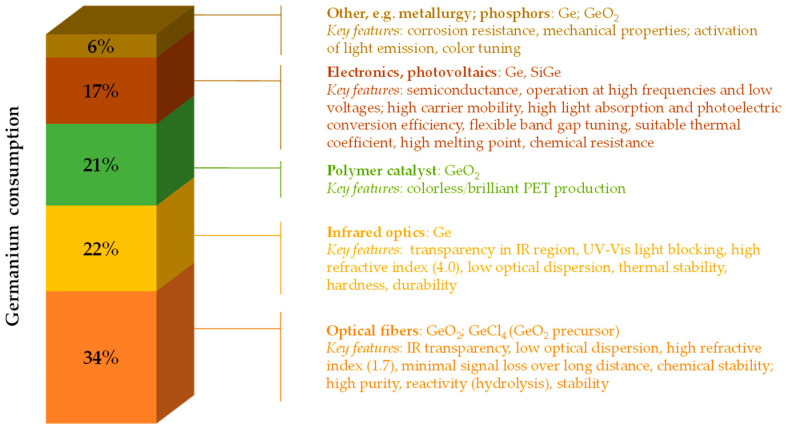
Distribution of global germanium consumption by end-use (in 2022 [7]) with key properties of germanium forms for specific applications.

Germanium is a historically important semiconductor and a pioneering material in the development of modern electronics, playing a key role in the construction of the first diodes and transistors for amplifying and rectifying electrical signals [1]. Today, germanium and its main compounds, such as germanium oxide GeO_2_, germanium chloride GeCl_4_, and SiGe alloys, are essential for advanced high-tech technologies and energy-related applications [2,3,4,5,6], particularly in electronics, photonics, optoelectronics, and photovoltaics (Figure 1).

Global production of refined germanium is relatively small but has fluctuated while generally increasing over the last twenty-five years, reaching 140 t in 2021 [8] (the latest available data). China dominated the market, supplying 93.5% of the metal (2022), followed by Russia (3.6%), Japan, and the USA (each at 1.4%) [9]. Future demand for germanium is expected to grow, driven mainly by advancements in artificial intelligence (enhancing high-performance microchips and photonic components), the development of 5G/6G communication systems (e.g., high-speed radio and optical networks), increasing demand from consumer electronics (e.g., optical fibers, IR cameras, optical components), the military and defense sectors (e.g., IR sensors, night vision systems, optical technologies), and multi-junction solar cells (e.g., for space applications) [10,11,12,13]. However, the supply situation has recently become more complex after China imposed export restrictions on germanium since August 2023, increasing risks of delivery continuity, and forcing changes to the germanium supply chains [14,15,16,17,18,19,20]. As a result, China’s reported germanium metal exports for the year through August 2024 dropped by about 55% (to 16.7 t), compared to the same period in 2023, with most of the exports (65%) directed to the European Union (Belgium, Germany) [14]. In 2024, Belgium partnered with DR Congo to process germanium from mining residues, aiming for an annual production of 30 t Ge, potentially supplying 30% of global demand [17,18]. Meanwhile, the price of germanium surged to 4150 USD/kg in March 2025, showing an increase of about 75% compared to January 2023 [21].

Although germanium is not a rare element [22,23], it is scarce and widely dispersed in natural sources (Table 1). Its common minerals include sulfides (i.e., argyrodite Ag_8_GeS_6_, briartite Cu_2_(Fe,Zn)GeS_4_, renierite (Cu,Zn)_11_Fe_2_(Ge,As)_2_S_16_, germanite Cu_13_Fe_2_Ge2S_16_) and hydroxides (i.e., stottite FeGe(OH)_6_) [22]. Germanium does not form its own specific ores, but occurs in minor or trace amounts in silicate, sulfide, and oxide minerals, and is recovered commercially as a by-product mainly from Zn–Pb and Zn–Pb–Cu–(Ag) ores [22,23,24,25,26]. Due to its high affinity for organic matter, germanium is often found in carbonaceous materials, including coals [10,22,23]. It is estimated [12,25,27] that 60–75% of global germanium production comes from sphalerite-rich zinc ores, while the remainder originates from coal fly ash. Other potential sources of germanium include refining residues from zinc, lead, copper, silver, gold, and nickel metallurgy (nearly 69,600 t Ge), as well as by-products of bauxite processing (about 57,200 t Ge) [12].

Germanium reserve data are not widely publicly reported, making it difficult to assess the availability of reserves [14]. Patel and Karamalidis [10] estimated that worldwide zinc ores (sphalerite) can contain 7000–13,000 t Ge, while coals can hold 25,000–112,000 t Ge. In turn, Frenzel et al. [23] evaluated that at least 119,000 t of recoverable germanium exists within proven reserves, with about 7000 t in zinc ores and about 112,000 t in coal, at grades exceeding 100 ppm and 200 ppm, respectively. They also calculated that at least 400,000 t Ge (50,000 t in zinc ores and 390,000 t in coals) should become recoverable in the future. Recently, Sverdrup and Haraldsson [12] estimated the total global extractable germanium potential at 342,550 t, out of a total geological presence of 5,569,000 t. However, their simulations indicated that only 45,000 t of germanium will be successfully extracted by the year 2200. These assessments do not account basically for recovering germanium from secondary sources [25,26,27,38,39,40], such as germanium scraps from manufacturing (or spent) optical fibers, IR lenses and windows, germanium wafers, etc., although simulations [12] predict a sharp increase in the recycling supply before 2050. While some countries (e.g., China [20], Belgium [40], USA [14]) have the capability to recycle these materials, it is presently carried out to a marginal extent.

Due to high demand, limited production and uncertainty regarding supply continuity, germanium has been classified as a critical and/or strategic material by several countries (regions), including Australia [41], Canada (Ontario) [42], DR Congo [43], the European Union [16], India [44], Indonesia [43], Japan [43], the United States [45]. In the long term (2040–2050), it is expected that germanium will face soft scarcity, followed by physical shortages [12,13]. On the other hand, coals represent an important source and reserves of germanium [10,12,13,25,26], and in some regions (the United Kingdom, former Czechoslovakia, the former USSR, Japan, China), germanium was recovered from coal fly ashes already in the 1950s and 1960s [46,47]. Therefore, the aim of this paper is to explore the latest achievements and methods for recovering germanium from such non-ore sources. This review examines the characteristics of coal and coal fly ash from combustion and gasification, focusing on their germanium content, modes of occurrence, and hydrometallurgical strategies for metal recovery, both industrially implemented and laboratory tested.

## 2. Germanium in Coals

### 2.1. Global Reserves

Coal was identified as a potential germanium source already in the 1930s [48]. Today, it has emerged as an important source of germanium. Nearly two-thirds of China’s total primary germanium production comes from germanium-bearing coal (113 t Ge in 2019) [13], while national reserves are estimated at 6670 t Ge (in coal ash) [10]. Russia has the largest share of active and potential coal germanium resources of 17,500 t [10] in several germanium-bearing coal beds [47]. The USA does not produce germanium from coal, but its coal reserves are estimated to contain approximately 1,703,000 t of germanium [49]. In fact, the actual recoverable amount is likely much lower, as only 16% of samples in the US Geological Survey Coal Quality Database (COALQUAL 3.0) have been reported to contain 10–235 ppm Ge [10]. There is a lack of estimated data on germanium resources in coal from other regions of the world.

Simulations indicate the growing contribution of coal to germanium production over time [12]. Models predict that by 2200, coal could supply about 16,000 t Ge, a quantity comparable to the 17,000 t Ge provided by zinc sulfide ores. Frenzel et al. [23] estimated that extractable germanium from hard coals (bituminous, anthracite) and brown coals (lignite, subbituminous) amounts to 287,000 t and 218,000 t, respectively. Worldwide germanium reserves in brown coal (based on Chinese and Russian reserves of coal with at least 200 ppm Ge) were estimated at 104,000 t. However, similar calculations could not be made for hard coals, as no mine was known to produce coal with such high germanium concentrations, although the existence of such mines was predicted by the used dataset.

### 2.2. Cut-Off Grade

The occurrence of germanium in coal does not necessarily equate to its actual and economically viable recovery, as the recovery level is directly related to the cut-off grade. Frenzel et al. [23] classified coals into three groups based on germanium content: 8–32 ppm, 32–200 ppm, and above 200 ppm, indicating different levels of extractable germanium at 13%, 33%, and 100%, respectively. A value of 300 ppm Ge in coal ash has been recommended as a cut-off grade [50]. Lin et al. [49] estimated that this threshold corresponds to 41 ppm of germanium in the coals, assuming an average ash yield of about 13%. For Chinese coals, 100 ppm has been reported as a minimum industrial criterion [51]. Shpirt et al. [52] proposed minimum concentrations of germanium in coal and fly ash of 30 ppm and 100 ppm, respectively. In turn, Salikhov et al. [53] recommended 10 ppm Ge for coals of practical interest.

### 2.3. Concentration

On a global scale, average germanium concentration in coals is 2.2 ppm [54], though certain local deposits can contain much more than 1000 ppm Ge. An analysis of almost 1600 coal samples from over fifty countries, compiled in the World Coal Quality Inventory database (USGS) [55], shows the mean germanium content at 5.2 ppm (on a dry, whole-coal basis). Literature data [46,47,48,49,50,51,52,53,54,55,56,57,58,59,60,61,62,63,64,65,66,67,68,69,70,71,72,73,74,75,76,77,78,79,80,81,82,83,84,85] indicate regional variations in germanium concentrations among coal deposits worldwide (Table 2), yet there is no consensus on which type of coal serves as a better germanium source. Frenzel et al. [23] considered that both low-rank and hard coals could be equally significant sources of germanium. In turn, Lin et al. [49] established that US bituminous coals may offer greater potential for future germanium recovery due to their generally higher germanium concentrations compared to low-rank brown coals (lignite, subbituminous). The latter is consistent with general observations that germanium is weakly enriched in hard coals globally compared to brown ones [54,73]. However, other reports [46,47,63,66,67,68,69] indicate that the germanium concentration in some lignite and subbituminous coals is two orders of magnitude greater than the global average concentration.

There are three giant coal deposits in the world that are currently being mined as raw material for germanium extraction. These are located in China (Lincang, Yunnan Province, and Wulantuga, Inner Mongolia) and Russia (Spetsugli, Primorski Krai) [46,47,57,69,74,75]. All are brown coal deposits, low-rank and with low calorific value, yet they contain abnormally high concentrations of germanium, with reserves exceeding 10,000 t [47,50].

Germanium concentrations in Lincang’s coal deposits reach up to 2500 ppm, with an average of about 850 ppm, with germanium reserves over 1000 t [46,57,58]. Metal is primarily extracted from three high-germanium coal mines (Dazhai, Meiziqing, Zhongzhai), having total reserves of about 730 t Ge. Wulantuga coal deposits contain 22–1894 ppm Ge [76,77,80], with an average of 273 ppm [46]. The high-germanium coal deposit is located in the Shengli Coalfield, where the major host seam has an average thickness of about 16 m and the deposit can contain 244 ppm Ge, with its reserves reaching up to 1800 t [59]. Remarkably, the probably largest germanium-bearing coal deposit in the world is located in China’s Wumuchang (Yimin Coalfield, Inner Mongolia), although it is not currently mined. Its estimated reserves are 4000 t Ge, with gallium concentrations reaching up to 470 ppm, often in a range of 50–200 ppm [46], although 43 ppm as an average value is reported [60,65]. Other germanium-bearing coals can be found also in Shanxi Province (Datong Coalfield, Yanzishan Mine, mean 12 ppm Ge in hard coal [81]), Anhui Province (Huainan Coalfield, mean 13 ppm Ge in bituminous coal [82]), or Henan Province (Yuzhou Coalfield, mean 33 ppm Ge in hard coal [83]).

The Spetsugli high-germanium coal deposits, with average concentrations of 450–514 ppm Ge and reserves of 1015 t Ge, are located in the Pavlovsk Coalfield, Russian Far East [47,57,64]. Notably, the highest recorded mean germanium concentrations in lignite coal samples from this region reached 1027 ppm [69], 1249 ppm [74], and even 1600–2540 ppm [75]. Other germanium-rich subbituminous coal deposits are located in Primorski Krai (Bikinsk: 300 ppm Ge and 2600 t; Shkotovsk: 1040 ppm Ge and 880 t, Rakovsk: 230 ppm and 280 t), Sakhalin (Novikovsk: 700 ppm Ge and 1665 ppm Ge), Transbaikalia (Tarbagataisk: 53 ppm Ge and 340 t) [47].

USA’s germanium-bearing coals with 10–100 ppm Ge are mainly located in Eastern (mean 4.2 ppm Ge) and Interior (mean 16.3 ppm Ge) coal provinces [10,70,78,79]. A potential source of extractable germanium was identified in North Dakota (Church deposit, lignite, 40–70 ppm Ge, 165 t reserves), while the highest germanium content was found in Montana (Fort Union, subbituminous coal, 235 ppm Ge) [10].

### 2.4. Occurrence Modes

Among the metals detected in coals, germanium exhibits rather unusual characteristics. In many coalfields, coal seams located at the edges of basins contain higher germanium concentrations than those in the center [22,60,77,78,80]. Germanium enrichment is often observed in the upper and/or lower marginal zones of coal beds [46,58,70,74,75,78,79,84], with higher concentrations found in thin seams compared to thick ones [77,78,85]. These phenomena (known as Zilbermint's law) have been attributed to the infiltration of germanium-enriched solutions from surrounding sediments into the coal bed [47,58,77,80,85].

Germanium is classified as a chalcophile, lithophile, organophile, and siderophile element [22,29]. It exhibits one of the highest affinities for organic matter among elements commonly found in carbonaceous sediments [22], making the organic association the primary mode of germanium occurrence in coals [58,59,63,65,66,67,72,73,75,78,86,87,88,89,90,91]. This is further supported by a negative correlation between germanium content and ash yield [59,60,81,82]. Spears and Zheng [73] observed a sudden increase in germanium content from below 1 ppm to 3–10 ppm in UK bituminous coals with volatile matter contents greater than 30%. Notably, germanium in lignites is often associated with elevated concentrations of other elements such as arsenic, tungsten, and beryllium [63,75,86].

It is estimated [66] that 75–96% of the total germanium in high-germanium lignites is organically bound, typically in complex humates (chelates), with preferential enrichment observed in huminites [67,86]. Bo et al. [88] performed sequential extraction of lignite (171 ppm Ge) as well as sink (15 ppm Ge) and float (365 ppm Ge) fractions after lignite gravity separation (in a liquid with a density of 1.8 g/cm^3^). Organic matter-bound germanium represented 65% in the raw coal and 84% in the float fraction, with both containing 4–5% of water-soluble germanium forms. On the other hand, the sink (mineral) fraction contained 69% of germanium in the form of carbonates and only 21% in organic form. Klika et al. [89] showed that data on the organic affinity of germanium in lignite can vary depending on the method used. Sequential extraction method yielded a value of 28%, while float-sink data combined with micropetrographic analysis resulted in a value of 93%. The sequential extraction method assumes complete dissolution of selected minerals (carbonates in HCl, clay minerals in HF, and sulfides in HNO_3_), releasing germanium into solutions. However, this approach incorrectly assumes a constant ratio between the percentages of the extracted element and the matching dissolved mineral across all coal fractions. Consequently, more realistic data were obtained from element affinity calculations based on sink-float data, also taking into account correlation analyses and direct detection of germanium in lignite fractions through microprobe analysis.

Etschman et al. [63] suggested that germanium accumulation in lignite occurs through the complexation of inorganic aqueous species (i.e., tetrahedral Ge(OH)_4_) with organic ligands (in distorted octahedral coordination with oxygen), forming strong bonds between Ge(IV) ions and the organic matter, rather than through the reduction in Ge(II) species. In turn, Wei and Rimmer [67] concluded that germanium species are weakly bonded to the organic matter in lignite (as chelates), as these could be easily removed by a mixture of acids (HCl-HF). Wei et al. [87] found experimentally that phenolic hydroxyl groups are likely the primary bonding sites for germanium ions. More detailed calculations (density functional theory) [90] showed that Ge(IV) ions exhibit a strong binding ability to oxygen functional groups and can form thermodynamically stable half-sandwich complexes with aromatic structures in coals of different ranks. Recently, Mu et al. [91] proposed a molecular formula for germanium-rich coal as C_166_H_162_N_2_O_32_, where Ge(IV) ions are immobilized through coordination bonds with the oxygen atom in the carboxyl group –COOH and the carbon atom in the pyrrole ring, while being distant from the nitrogen atom in the pyrrole ring. They also explored the organic modes of germanium occurrence in coals of different ranks. Oxygen-containing functional groups and aromatic rings show a strong binding affinity for Ge(IV), enhancing its enrichment in lignites. With coal rank changing to bituminous, the reduction of oxygen-containing functional groups, like –COOH and –C=O, and the relatively low condensation of aromatic rings decrease the number of available binding sites for metal ions, leading to a decline in binding ability and lower germanium concentration. Highly condensed aromatic rings in anthracite provide binding sites for Ge(IV) ions, and the strong binding affinity of these rings could facilitate germanium enrichment. However, the low content of oxygen-containing functional groups in anthracite diminishes their influence on the organic modes of germanium occurrence.

The significance of mineral matter in germanium distribution in coals appears to be minimal. However, its role becomes more prominent in partings and host rocks, which are characterized by high germanium content and low organic matter content [22,66,75,85]. In most coal deposits, small amounts of germanium are associated with sulfide minerals, primarily pyrite [63,70,86], or with clay minerals [70]. Arbuzov et al. [75] identified aluminosilicates (0.15–0.4% Ge), various ferrous minerals (0.1–6.2% Ge) like hydrogoethite, goethite, and jarosite, or polymineral phases as germanium-bearing minerals in Spetsugli’s brown coals.

### 2.5. Germanium Recovery

Germanium recovery is not typically carried out directly from coal due to its low concentration, uneven distribution in coal seams, and consequently, the high cost of extraction, making the process unprofitable. However, it is worth noting the cooperative exploration model of the Wulantuga coal–Ge deposit developed by Li et al. [92] to improve strategic coordination as well as economical and effective exploration techniques.

**Figure 2:**
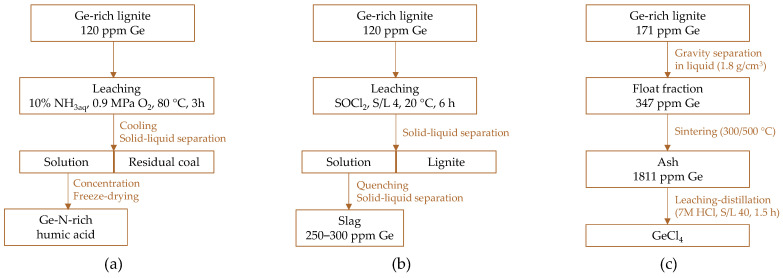
Schemes of direct germanium recovery from lignite, based on ref. (**a**) [94], (**b**) [95], (**c**) [88].

Lapidus et al. [93] reported that hydrometallurgical treatment does not achieve complete germanium recovery, even when using aggressive leaching agents such as a mixture of HF and HCl. This method resulted in a 49% recovery rate from bituminous coal and 83% from lignite. Comparable results of 83% were obtained with alkaline leaching of brown coal, but the recovery rate increased to 87% when oxidized coal treated with H_2_O_2_ was used. The relatively weak binding of Ge(IV) in the form of chelates in lignite has recently stimulated studies on direct leaching. Zhang et al. [94] proposed a method for the co-extraction of germanium and humic acid from germanium-rich lignite (Figure 2a). They conducted ammonooxidation of lignite in an autoclave (10% NH_3aq_, S/L 10, 0.9 MPa O_2_, 40–200 °C, 1–5 h). Under optimal conditions (80 °C, 3 h), almost 27% of germanium was recovered, and the total extraction rate after three sequential ammonooxidation stages reached 42%. This approach improved the recovery of valuable nitrogen-rich humic acid, compared to direct combustion, which can be used as a biofertilizer or N-doped carbon material. The idea of simultaneous germanium and lignite recovery was further developed by using thionyl chloride, SOCl_2,_ as a leaching agent [95]. Under optimal conditions (L/S 4, 20 °C, 6 h), the recovery of both components exceeded 95%. This process preserved the matrix structure and main composition of lignite while simultaneously enabling high germanium recovery with a low carbon footprint compared to traditional treatment (Figure 2b).

Bo et al. [88] developed a three-stage process for germanium recovery from lignite (Figure 2c). Raw coal particles were separated by gravity in a liquid medium (1.8 g/cm^3^) to obtain a germanium-enriched float fraction. This fraction was then sintered at low temperatures (300/500 °C) to accumulate germanium in the ash. The next stage involved leaching GeO_2_ from the ash with HCl (6–9 M, L/S 10–50, 25 °C, 0.5–2 h), followed by the distillation of GeCl_4_, resulting in over 90% germanium recovery.

Recently, pyrolysis has been proposed as an alternative method for germanium volatilization and enrichment. Cai et al. [96] demonstrated that heat treatment (300–900 °C) of bituminous coal under a nitrogen atmosphere affects germanium distribution. In the raw material, germanium was predominantly present in the sulfide fraction, followed by the carbonate and residual forms. As the pyrolysis temperature increased, the germanium distribution changed due to the decomposition of sulfides, leading to its accumulation in the carbonate and residual fractions. Liu et al. [97] reported that the extraction of germanium from lignite through pyrolysis under a reducing atmosphere (CO, CH_4_, H_2_, H_2_S released from decomposed organic matter) involves a series of reactions forming volatile species like GeO, GeS, and Ge. These compounds subsequently condensed in the low-temperature zone, producing a germanium-rich product containing 25.5% Ge, primarily as sulfide.

## 3. Germanium in Coal Gangue

Coal gangue is a large-volume waste product, accounting for 10–30% of raw coal production, generated during coal mining and washing [98]. It is a black-gray rock with low carbon content and poor calorific value. This waste is typically stockpiled in heaps near mines, leading to environmental pollution due to weathering and water leaching. However, coal gangue has been recognized as a potential source of critical elements such as lithium [99] and gallium [100].

Wang and Wang [101] investigated coal gangue from major Chinese coal mines in Shaanxi Province and identified germanium as a potentially extractable element. They analyzed samples from the roof, parting, and floor layers of coal deposits (Table 3). The weighted average germanium concentration in coal gangue was 2.2 ppm, which is comparable to the global average in coals and close to the average concentration in Chinese coal. Based on China’s annual coal gangue production of 800,000 t, a crude germanium reserve of 1221 t was calculated. Considering the total cumulative discharge of coal gangue (4.5 billion tons), the estimated reserves could reach 6867 t Ge.

Wu et al. [102] analyzed the distribution of germanium in five particle size fractions (50–200 mesh) of coal gangue. They observed a slight increase in germanium concentration in smaller particles, ranging from 10.6 ppm in the 50-mesh fraction to 15.7 ppm in the 200-mesh fraction. No distinct mineral composition was identified for specific fractions, and germanium was primarily incorporated into the glassy phase, embedded within the Si–Al lattice, or adsorbed onto mineral surfaces. Detailed analysis revealed its association with calcite, iron–manganese oxides, pyrite, and organic matter. Sequential extraction analysis confirmed that most germanium was present in the residual (60–69%), sulfide/organic-bound (22–26%), and metal-oxide (7–12%) fractions, while only trace amounts were found in the ion-exchangeable (0.2–0.3%) and carbonate (1.2–1.4%) fractions, regardless of particle size.

Currently, no methods for germanium recovery from coal gangue have been developed.

## 4. Germanium in Coal Combustion Ashes

### 4.1. Concentration

The combustion of coal and coal gangue in coal-fired power plants generates waste materials such as fly ash, bottom ash, and boiler slag [103]. Coal fly ash constitutes the majority (40–90%) of these residues and is captured from flue gas as a fine-grained, powdery material with micrometer-sized particles. Main components of the fly ash are SiO_2_ (31–60%), Al_2_O_3_ (11–55%), Fe_2_O_3_ (1.5–19%), CaO (up to 30%), as well as K_2_O, Na_2_O MgO, SO_3_, each up to 2–5% [60]. Bottom ash [103] accounts for 10–20% of the total waste and consists of agglomerated, porous particles in the millimeter size range. These particles are too large to be carried into the flue gasses and therefore accumulate at the bottom of the coal furnace. Main components of the bottom ash are SiO_2_ (45–61%), Al_2_O_3_ (19–25%), Fe_2_O_3_ (about 6%), CaO (up to 18%), as well as K_2_O, Na_2_O, MgO, each up to about 1%. Boiler slag consists of granular, hard, glassy particles, typically a few millimeters in size. It is produced exclusively in specialized boilers and represents a major component of their by-products (70–85%). Main components of the boiler slag are SiO_2_ (32–64%), Al_2_O_3_ (up to 43%), Fe_2_O_3_ (up to 9%), CaO (up to 20%), as well as K_2_O, Na_2_O MgO, SO_3_, each up to 1–2% [60]. Moreover, coal samples are typically burned slowly under laboratory conditions to produce coal ash for analytical purposes [104].

During coal combustion, germanium is released from coal and redistributed among solid residues and flue gas. Zhou et al. [105] found that the distribution of germanium during coal combustion is as follows: 62% in fly ash, 22% in flue gas, and only 16% in bottom ash (under laboratory conditions). Similarly, during combustion of coal gangue, 55–80% of germanium accumulates in ash, while 20–45% in the gaseous phase [102].

Germanium is classified as a Group 2 element according to the classification of trace elements based on their behavior during combustion and gasification [106]. This group includes volatile elements (e.g., As, Cd, Ga, Pb, Sb, Zn, etc.) that vaporize during combustion and subsequently condense onto particle surfaces in the flue gas stream during cooling. These elements preferentially condense on smaller fly ash particles due to their higher surface-to-volume ratio. As a result, germanium is more enriched in fly ash and depleted in bottom ash (Table 4). For example, Zhou et al. [105] found that fly ash contains at least 60% more germanium than bottom ash. Moreover, the concentration of germanium increases as the fly ash particle size decreases. Dai et al. [57] reported a threefold higher germanium concentration in fine-grained fly ash (i.e., collected from baghouse filter; 14,870 ppm Ge) compared to coarse fly ash (i.e., collected from electrostatic precipitator; 4731 ppm Ge) from high-germanium Wulantuga coals, while an almost 22-fold higher germanium concentration was observed in fine particles (24,600 ppm) compared to coarse particles of fly ash (1120 ppm) from Spetsugli high-germanium coals. Similarly, Lanzerstorfer [107] observed variations in germanium content across five size fractions of coal fly ash (13 ppm Ge, mass median diameter d_50_ = 17.6 μm) collected from the off-gas de-dusting system of a power plant using hard coal. An increase in element concentration was reported, from 2 ppm for the largest particles (d_50_ = 43.2 μm) to 17 ppm for medium-sized particles (d_50_ = 9.7 μm), reaching 29 ppm in the smallest particle fraction (d_50_ = 2.2 μm).

The average germanium concentration in coal ashes is commonly established as 15 ppm, though it is slightly higher in hard coal ashes (18 ± 1 ppm) than in brown coal ashes (11 ± 1 ppm) [54]. Admakin [68] showed that lignite ash contains more germanium than coal ash, as isolated lignite deposits in coal beds are typically rich in germanium. However, exceptionally high concentrations, on the order of a few percent, have been reported sporadically. For example, lignite ash from the Atlantic Coastal Plain (USA) has been found to contain 2.0–7.5% Ge [127].

### 4.2. Occurrence Modes

During the combustion of coal and coal gangue, germanium becomes enriched in ashes while simultaneously undergoing transformation through high-temperature reactions. These processes facilitate its volatilization and modify its associations with ash components due to the decomposition of organic compounds, which serve as the primary host of germanium in coals. Frandsen et al. [128] investigated the behavior of germanium during the thermal conversion of coal under equilibrium conditions. Based on thermodynamic calculations, they showed that under standard oxidizing conditions, crystalline GeO_2_ is stable up to 700 K (427 °C), while in the range of 800–2000 K (527–1727 °C), the equilibrium form gradually changes with increasing temperature, from gaseous GeS as the major germanium species at 800 K to gaseous GeO at 2000 K.

**Figure 3:**
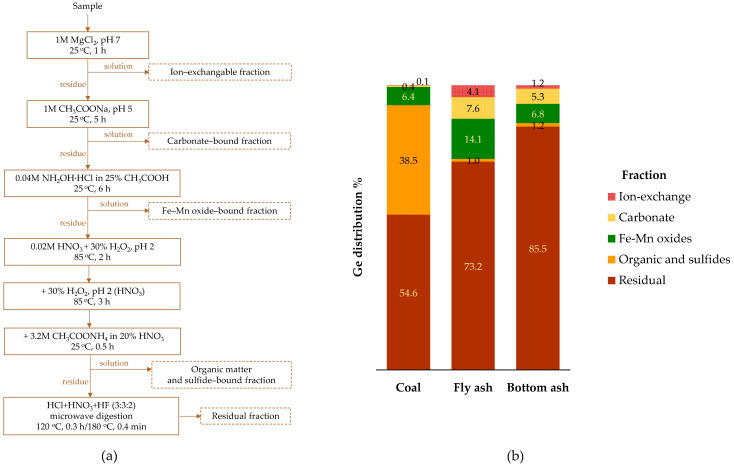
Scheme of Tessier sequential extraction procedure (**a**) and occurrence modes of germanium in raw coal and coal combustion ashes (**b**), based on ref. [105].

Zhou et al. [105] analyzed the modes of germanium occurrence in fly and bottom ashes produced during coal combustion (500–1000 °C under laboratory conditions) using the sequential extraction method (Figure 3a). They showed that germanium’s association with organic matter in feed coal decreases from approximately 38% to just 1% in fly and bottom ashes (Figure 3b). Simultaneously, there was an increase in germanium accumulation, primarily in the residual and carbonate fractions of the ashes. Additionally, a small proportion appeared in an ion-exchangeable form, which was absent in the feed coal. In fly ash, the proportion of germanium in oxide form was also observed to be twice as high as in the other two materials. A comparable distribution of germanium, with dominant accumulation in the residual phase (70%) and lower amounts in the carbonate and oxide fractions (both representing about 25%), was also reported for fly ash collected from a pulverized coal furnace in a power plant [129].

A similar sequential extraction procedure was used by Wu et al. [102] for coal gangue combusted at different temperatures (600–1000 °C under laboratory conditions). They observed that most of the germanium accumulated in the ash, with 78–81% of germanium present in the residual fraction, incorporated into the Al–Si lattice of glass. The distribution of germanium among other fractions—such as metal oxide, carbonate, ion-exchangeable, and organic/sulfide—was 8–9%, 6.5–9.1%, 2.3–2.8%, and 1.5–1.8%, respectively.

Dai et al. [57] determined the main germanium compounds in fly ashes derived from the combustion of high-germanium coals at the germanium recovery plants in Wulantuga and Lincang. They found that particles of secondary fine-grained carbon (unburnt) embedded fine-grained Ge-bearing mineral phases. Germanium oxides (mainly GeO_2_) were the major germanium carriers in the fly ash. Other Ge-bearing phases found included glass, calcium ferrites (Ca_n_Fe_n_(Ge,W)_n_O_x_, (Ca,Mg)_n_(Fe,Mn)_n_(AlSi)_n_(Ge,W)_n_O_x_), calcium germanate Ca_2_GeO_4_, solid solutions of germanium in silica SiO_2_, and possibly elemental germanium or Ge(Ge-W) carbide. Notably, previously unknown complex oxides such as (Ge,As)O_x_, (Ge,As,Sb)O_x_, (Ge,As,W)O_x_, (Ge,W)O_x_, and (Ge_9_,Pb)O_20_ were identified. They also observed that some portion of germanium occurred as adsorbed species in different types of unburnt carbon in the ash particles.

### 4.3. Physical Preconcentration

The occurrence modes of germanium and its strong tendency to accumulate in finer particles of fly ash during coal combustion are of great significance for selecting effective and cost-efficient recovery technologies. Therefore, physical properties such as particle size, density, magnetic and electrostatic properties, and wettability behavior of the solid particles can serve as feasible criteria for element preconcentration using simple separation methods.

**Figure 4:**
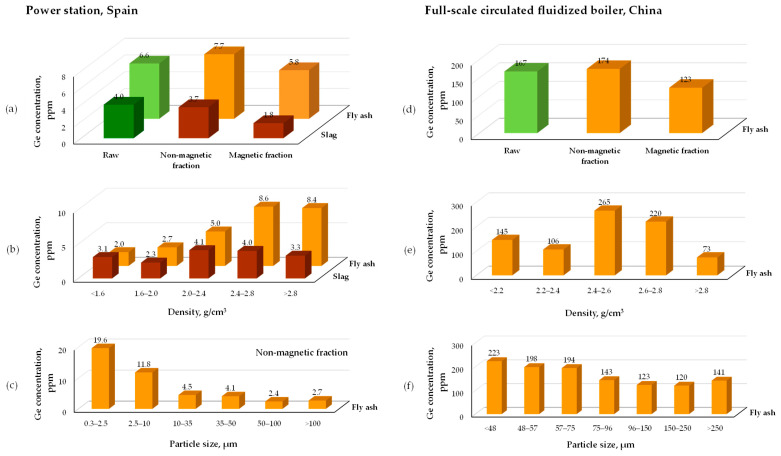
Germanium concentration in slag and/or fly ash fractions of different physical characteristics: (**a**,**d**) magnetic, (**b**,**e**) density, (**c**,**f**) size (only non-magnetic fraction); (**a**–**c**) based on ref. [122], (**d**–**f**) based on ref. [130].

Querol et al. [122] compared the behavior of germanium during the separation of low-germanium fly ash (6.6 ppm Ge) and slag (4 ppm Ge), taken from the Spanish power station, into different fractions. They classified germanium as an element that is more strongly enriched in fly ash, showing an affinity for the aluminosilicate nonmagnetic phase, particularly in the size fraction below 10 μm and density fractions with a minimum of 2.4 g/cm^3^ (Figure 4a–c). In fact, the fractionation of germanium was particularly pronounced in the size segregation of the non-magnetic portion of fly ash, with an enrichment factor of about 2.8. The dominant accumulation of germanium in the nonmagnetic fraction of industrial fly ash was further confirmed by other studies [129,130,131].

A similar distribution analysis was performed by Zhou et al. [130] for germanium-rich fly ash (167 ppm) collected from a Chinese power plant. They observed a similar tendency in germanium distribution between magnetic and non-magnetic fractions, with the highest accumulation in the particle density fraction of 2.4–2.8 g/cm^3^. However, they noted a minimum germanium concentration in the 150–250 μm particle size fraction (Figure 4d–f). Additionally, a strong enrichment of germanium in the glass phase (235 ppm Ge) compared to other mineral phases (90 ppm Ge) was found. The authors concluded that germanium can be physically preconcentrated in moderate-density fractions, as it is associated with mullite and the glass phase.

In turn, Li et al. [129] observed practically no germanium enrichment across the density fractions of fly ash (9 ppm Ge) collected from a power station (pulverized coal furnace), ranging from below 1.8 g/cm^3^ to above 2.4 g/cm^3^, although the latter showed a slight enrichment factor of 1.06. Magnetic separation into five fractions yielded similar results. Better outcomes were obtained when analyzing size fractions, as the enrichment coefficient was higher than 1.0 for all fractions (except for the 106–150 μm fraction, which was 0.99) and reached the highest value of 1.22 for the smallest particles (below 45 μm). Thus, it was concluded that size separation has the greatest potential for germanium concentration from such fly ash.

There is no unequivocal separation method for germanium enrichment from coal fly ash, as the optimal approach depends on the unique characteristics of the ash, such as particle size distribution, density, and mineral composition. Consequently, the most effective technique must be selected based on the specific properties of the fly ash being processed.

### 4.4. Industrial Recovery

Although some processes for germanium extraction from coal ash were developed several decades ago [48,132,133,134], they are currently implemented in only two countries (China and Russia). The details of these technologies are not widely available, and the most recent literature data [46,57] dates back around ten years. The extraction plants are located in three regions and utilize high-germanium coals from the Wulantuga, Lincang, and Spetsugli deposits (Table 5). The coal is combusted in small and medium-scale power plants, producing fly ash, which is collected in electrostatic precipitators (coarse fraction) and baghouse filters (fine particle fraction). In these plants, germanium is extracted from fine-grained fly ash, which accounts for only 7–15% of the total coal combustion ash output.

**Figure 5:**
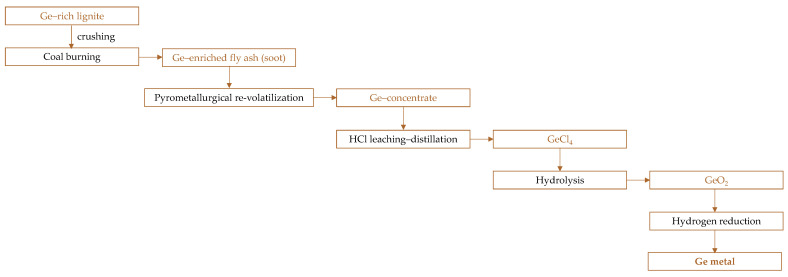
Scheme of germanium recovery from coal combustion fly ash, based on ref. [135].

**Figure 6:**
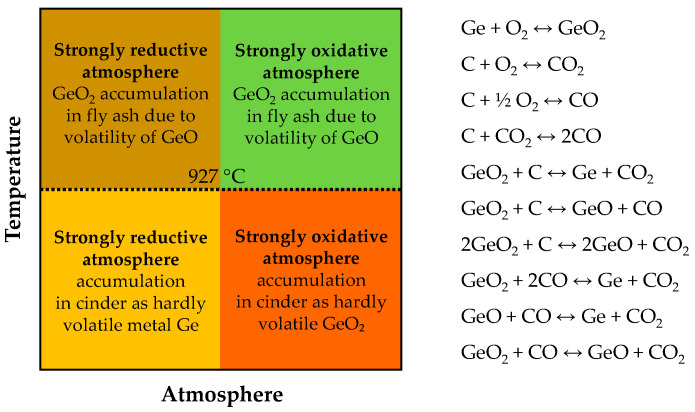
Germanium behavior during coal combustion, based on ref. [136].

Figure 5 presents a schematic representation of germanium recovery from coal in China. When germanium-bearing lignite is burned in an oxidizing atmosphere (air), germanium volatilizes as oxides and accumulates in fly ash (soot) [135]. This concentrate typically contains 1000–3500 ppm Ge. To maximize germanium recovery, combustion parameters (atmosphere, temperature [136]) must be adjusted (Figure 6), often at the expense of energy efficiency. In some cases, coal is burned exclusively for germanium extraction, making energy recovery unfeasible, although current approaches integrate thermal energy recovery for power generation and indoor heating. While conventional combustion methods achieve 40–60% germanium recovery, swirl furnaces can transfer up to 80–85% of the element into the soot.

It is worth mentioning that coal coking occurs at temperatures between 750 and 1050 °C, while semi-coking takes place at 450–500 °C, both in a reducing atmosphere consisting mainly of CO, H_2_, N_2_, CO_2_, and H_2_O [93]. The transfer of germanium to the gaseous phase is almost independent of the process temperature but increases with CO content. During coking, the sublimation of germanium as GeO does not exceed 20–30%, and the rest transitions into metallic form in the coke (Figure 6). During the cooling of gaseous products, GeO dissociates (either completely or partially) into GeO_2_ and Ge. Up to 75–85% of the germanium remains in the coke, and the rest, in the form of metal or dioxide, is collected on filters. Under industrial conditions, the gasses are not passed through filters, and germanium passes into tar or tar water.

Combustion at high temperatures (above 700 °C) leads to the formation of germinate, a solid solution of GeO_2_ and SiO_2_, which is highly resistant to leaching, requiring large amounts of acid while yielding low germanium recovery rates. As a result, a secondary enrichment process, known as the re-volatilization method, is employed (Figure 5). This pyrometallurgical process involves re-burning germanium-containing soot in a shaft furnace or blowers, producing a Ge-rich concentrate. However, since germanium must undergo volatilization twice, this method leads to higher losses and increased energy consumption, limiting total germanium recovery to 70–80%.

Following pyrometallurgical treatment, the material undergoes leaching with HCl (min. 7.8 M, in practice: 12 M to prevent salt hydrolysis [132]) to form GeCl_4_:

GeO_2_ + 4HCl → GeCl_4_ + 2H_2_O (1)

which is then distilled (GeCl_4_ boiling point is about 83 °C) to obtain a purified compound. The recovery rate of germanium in this step reaches around 80% [135].

Subsequent hydrolysis yields high-purity GeO_2_:

GeCl_4_ + (2 + n)H_2_O → GeO_2_∙nH_2_O + 4HCl (2)

which is reduced with hydrogen at 650–675 °C for a few hours to produce elemental germanium (and prevent GeO sublimation at 700 °C [132]):

GeO_2_ + H_2_ → GeO + H_2_O (3)

GeO + H_2_ → Ge + H_2_O (4)

To achieve an even higher purity level, the germanium metal undergoes zone refining at 1000–1150 °C. Finally, single-crystal germanium is obtained through the crystal-drawing process [135].

### 4.5. Laboratory Developments

Germanium recovery from coal combustion fly ash is often critically evaluated as an energy-intensive process with low yield and the release of toxic contaminants, such as arsenic compounds [10,22,25,31,40,52,93,135]. For this reason, alternative processes are being explored to achieve higher efficiency while also enabling the recovery of other valuable elements from fly ash in a more environmentally friendly manner.

Li et al. [137] briefly reviewed Chinese-language literature on the topic. They cited several publications indicating the possibility of germanium recovery with efficiency rates ranging from 85% to 95%; however, no details of the processes were provided. Similarly, Lapidus et al. [93] analyzed Russian literature, pointing out some achievements in this field. Therefore, only the types of processes studied are symbolically listed in Table 6 to highlight the different methods examined.

Chen et al. [138] investigated the influence of coal combustion temperature on the leachability of valuable metals from coal ash using HCl and NaOH solutions. They found that germanium is more readily leached by alkali than by acid, with higher efficiencies observed at a combustion temperature of 900 °C (~40% in NaOH, ~20% in HCl) compared to 1300 °C (~30% in NaOH, ~10% in HCl). However, no further details were provided regarding the behavior of germanium in both processes.

Zhang and Xu [139] investigated the vacuum reduction in coal fly ash (4986 ppm Ge), followed by chlorinated distillation to concentrate and produce pure germanium. They found that under optimal reduction conditions in a vacuum induction melting furnace (920 °C, 260 Pa, 16.6% coke powder as reductant), the germanium separation rate reached 93.4%. The reduction product contained 42.2% Ge in both elemental and dioxide forms, with impurities including 13% As, 2.9% Ca, 1.3% Zn, and 0.6% Pb. This product was then subjected to a chlorinated distillation process (8 M HCl, H_2_O_2_, L/S 7, 120 °C), followed by hydrolytic precipitation of GeO_2_ with 98% purity. Notably, 8% MnO_2_ was added to the HCl-H_2_O_2_ solution to remove arsenic (97%). The total germanium recovery for the integrated procedure was 83.5%.

**Figure 7:**
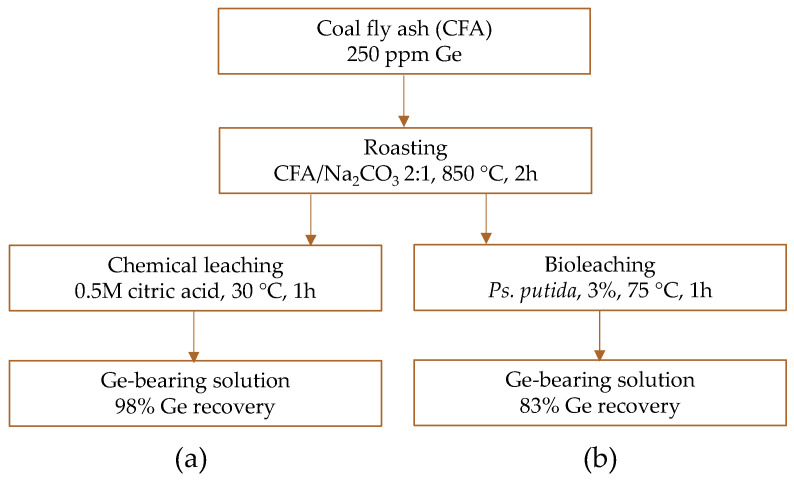
Scheme of germanium recovery from roasted coal combustion fly ash: (**a**) based on ref. [140], (**b**) based on ref. [141].

Razei et al. [140] developed a roasting–leaching process for germanium recovery (as well as vanadium and lithium) from fly ash (250 ppm Ge) taken from a coal power plant (Figure 7a). The salt roasting (NaCl, NaNO_3_, Na_2_CO_3_, Na_2_SO_4_; 850 °C, 2 h) was performed to decompose the aluminosilicate phase before leaching with organic acids (malic, citric, oxalic, acetic). It was found that the main parameter influencing germanium leaching was the type of leaching agent (60%), followed by the choice of roasting agent (23%), roasting time (9%), and acid concentration (8%), while the liquid-to-solid ratio had no significant effect. Citric acid and sodium carbonate were selected as the optimal leaching and roasting agents, respectively. Under optimal conditions, germanium recovery was 98%. Notably, the final germanium concentration in the solution was only 24.5 ppm.

Razei et al. [141] further improved the process by employing spent-medium bioleaching with *Pseudomonas putida* and *Pseudomonas koreensis*. Both bacterial species produce organic acids, mainly gluconic acid, oxalic acid, and citric acid, which were used for germanium leaching from both the roasting product (fly ash + Na_2_CO_3_) and untreated material. In all bioleaching experiments, germanium recovery increased with time up to 1–1.5 h, but then declined. The highest recovery rate of 83% was observed for the leaching with *Ps. putida* (Figure 7b), which produced organic acids at higher concentrations. Notably, biologically produced acids were more effective in germanium recovery compared to chemical leaching (58% recovery) using a synthetic mixture of the same three acids.

**Figure 8:**
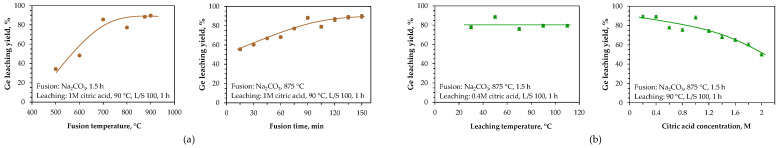
Effect of alkali fusion parameters (**a**) and leaching parameters (**b**) on germanium recovery from coal fly ash in citric acid solution, based on ref. [142].

Li et al. [142] developed a two-stage process involving alkali fusion and organic acid leaching of fly ash (11 ppm Ge) from a power plant. They found that, among various roasting agents (Na_2_CO_3_, NaOH, NaCl, CaO, CaCO_3_, (NH_4_)_2_SO_4_, CH_3_COONH_4_, NH_4_Cl) and leaching acids (citric, tartaric, lactic) as well as other process parameters (temperatures, times, L/S ratios), the optimal combination was sodium carbonate (fly ash/Na_2_CO_3_ 1, 875 °C, 1.5 h) and citric acid (0.4 M, 50 °C, L/S 125, 1 h). This resulted in over 90% germanium recovery. Although germanium recovery was not the primary focus of the study, the data provided allow for tracking the behavior of this element under different conditions of alkaline fusion (Figure 8a) and during leaching in citric acid solutions (Figure 8b). Notably, the study highlights the positive effect of increased temperature and fusion time of fly ash with sodium carbonate on the leaching rate, the negligible influence of leaching temperature on germanium extraction from the fusion product, and the decreasing leaching efficiency with increasing acid concentration. However, no interpretation was given for these observations.

Germanium, as a trace element in most coal combustion fly ashes, generates very diluted solutions during leaching. Although the concentrations of germanium ions are occasionally reported, their levels typically range in the tens of ppm (μg/L) in leachates. Considering the high concentrations of other ions (e.g., aluminum, iron, calcium, sodium) that are leached in significant amounts (up to tens of g/L), selective recovery of germanium ions becomes a challenge. For this reason, methods based on the specific properties of germanium ions are preferred, as they enable both separation and concentration effects. Unfortunately, the literature data dedicated to the final recovery of germanium species from coal combustion fly ash leachates are lacking yet.

## 5. Germanium in Coal Gasification Ashes

### 5.1. Concentration

**Figure 9:**
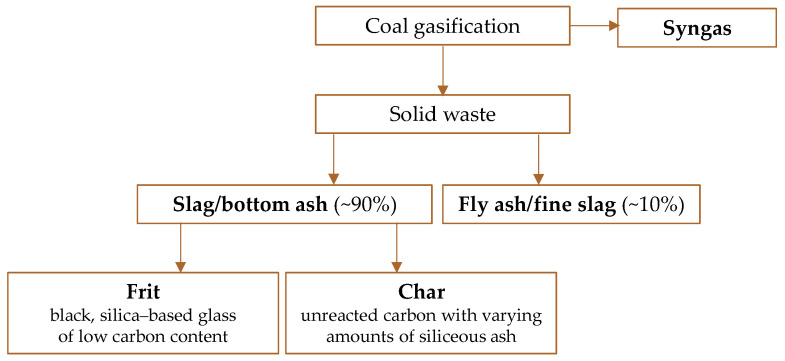
Main products of coal gasification.

Coal gasification converts coal into synthetic gas (syngas), which serves as either a fuel or a feedstock for chemical production [143]. The process also results in solid by-products, including ash and/or slag, along with unburned carbon (Figure 9). The primary inorganic components of these solid wastes are SiO_2_, Al_2_O_3_, CaO, MgO, and Fe_3_O_3_ in varying proportions [144,145,146,147]. Compared to conventional coal combustion, gasification produces less fly ash, as gasifiers typically operate at temperatures higher than the ash fusion point [143].

There is very limited data on germanium concentration in solid wastes from coal gasification, with most studies focusing on fly ash from the Integrated Gasification Combined Cycle IGCC plant in Spain [143,144,148,149,150]. These studies indicate a significant enrichment of germanium in the fly ash, with concentrations at least several times higher compared to the feed coal and slag, as shown by both laboratory experiments and industrial practices (Table 7).

The germanium content in coal gasification residues is influenced by the composition of the feed coal. Arroyo et al. [150] examined fly ash samples from a gasification plant in Puertollano, Spain, with germanium concentrations ranging from 174 ppm to 356 ppm across different operational periods. Their analysis revealed fluctuations of 40–60 ppm on a weekly and monthly basis, with significantly greater variations over extended periods—up to 94 ppm over several months and 200 ppm over years. These changes were related to the use of coals from newly mined seams, which contained different trace element levels than older seams. For example, germanium concentrations in coal stockpiles formed in 2008 ranged from 11 ppm to 20 ppm, whereas those in stockpiles dating back five to nine years were slightly higher, at 15–21 ppm. This resulted in slightly lower germanium concentrations in fly ash, with 174–268 ppm in “new” ash compared to 244–356 ppm in “old” ash.

**Figure 10:**
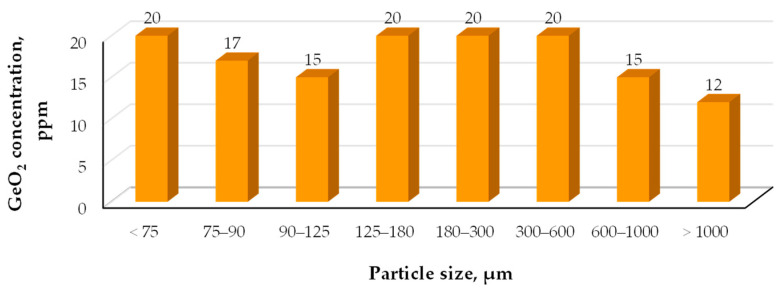
GeO_2_ concentration in different particle size fractions of coal gasification ash, based on ref. [146].

Saini et al. [146] analyzed fly ash from a commercial-scale fixed-bed gasification unit in India. The sample was separated into eight size fractions, ranging from a few microns to several millimeters, with more than 90% of the sample consisting of particles larger than 10 mm. The analysis of germanium concentrations in its oxide form revealed that medium-sized fractions had uniform GeO_2_ content (Figure 10). However, it should be noted that the analyses were conducted using XRF, and the oxide form was present at concentrations of only a few tens of ppm, which could be associated with significant measurement inaccuracies for such a multicomponent material.

### 5.2. Occurrence Modes

**Figure 11:**
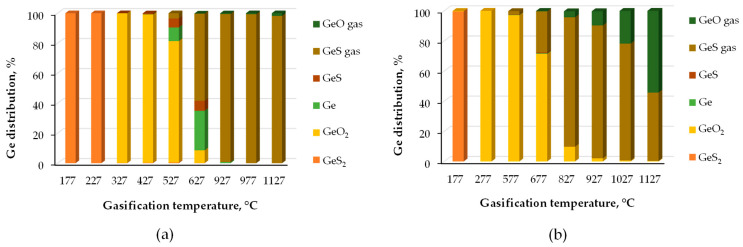
Temperature-dependent equilibrium germanium distribution during lignite gasification at different excess air coefficients: (**a**) 0.3, (**b**) 0.9; based on ref. [145].

Coal gasification generates solid waste products with a different composition compared to those from the coal combustion process. Shpirt et al. [145] analyzed the distribution of germanium during the gasification of lignite using thermodynamic methods. They found that the formation of specific germanium compounds (oxides, sulfides, and elemental germanium) in both condensed and volatile states depends on temperature and the assumed excess air coefficients (Figure 11). When the temperature reached approximately 930 °C, it was observed that 98% of the germanium present in the initial material underwent transformation into volatile GeS under conditions with lower excess air coefficients. However, when the air dosage was increased, germanium predominantly converted into a mixture of two gaseous compounds, GeS and GeO. At lower temperatures, germanium was predicted to accumulate in condensed phases, primarily in the forms of GeS_2_, GeO_2_, and elemental Ge, rather than remaining in the gaseous phase.

**Figure 12:**
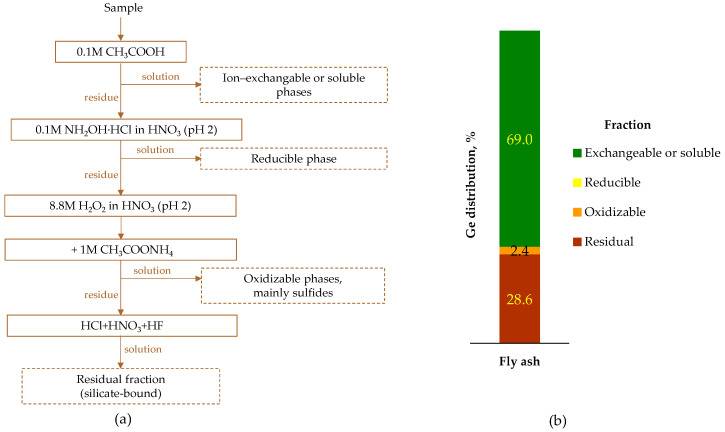
Scheme of BCR sequential extraction procedure (**a**) and occurrence modes of germanium in coal gasification fly ash (**b**), based on ref. [148].

Font et al. [148] analyzed fly ash from an IGCC plant to identify the real forms of germanium. For this purpose, BCR (Commission of the European Community Bureau of Reference) sequential extraction procedure (Figure 12) and advanced spectroscopic methods were used. They revealed that about 70–80% of the germanium existed as water-soluble species, including hexagonal-GeO_2_, GeS_2_, and slightly soluble GeS (sphalerite-bound [151]). The remaining germanium was associated with the residual aluminosilicate phase. Further phase composition studies [151] confirmed the presence of crystalline germanium phases, such as hexagonal-GeO_2_, GeS_2_, and GeSnS_3_.

### 5.3. Recovery

#### 5.3.1. Case Study

The recovery of germanium from coal gasification fly ash was initially developed for ashes produced at the Puertollano IGCC plant (Spain) and has undergone continuous advancements over the past two decades [143,144,148,149,150,151,152,153,154,155,156,157,158,159,160,161]. Due to the significant differences in the modes of germanium occurrence in combustion (~75% Ge in the residual fraction) and gasification (~75% Ge in the water-soluble fraction) fly ashes, leaching methods are more easily applied to the latter.

##### Leaching Step

Font et al. [151] investigated the water leachability of fly ashes (194–420 ppm Ge) with high (6%) and low (3%) CaO contents. They evaluated the effectiveness of leaching with pure water and three types of water (pH 7–9, with different ionic speciation) generated during coal gasification and gas cleaning processes at an IGCC plant, as well as the influence of leaching parameters (temperature, duration, L/S ratios). High germanium extraction rates (up to 53% for high-CaO ash and up to 84% for low-CaO ash) were achieved at room temperature (24 h). Increasing the temperature to 50–150 °C maintained the germanium leaching effect but reduced the amount of contaminants transferred into the solution, allowing for a shorter leaching time. The optimal leaching conditions (90 °C, L/S 5, 6 h) allowed for 66–86% Ge recovery, regardless of the water type used. In turn, Arroyo et al. [150] compared the germanium leachability from fresh (shortly after ash generation) and aged (a few years old) fly ash samples. Since the fly ash consisted mainly of a glass phase (97%), it was hypothesized that aging could promote the conversion of sulfide/arsenide species to sulfate/arsenate species with partial crystallization of the Al–Si glass matrix. However, water leaching tests (50 °C, S/L 5, 2–24 h) showed practically no aging effect on germanium extraction (25–78%).

**Figure 13:**
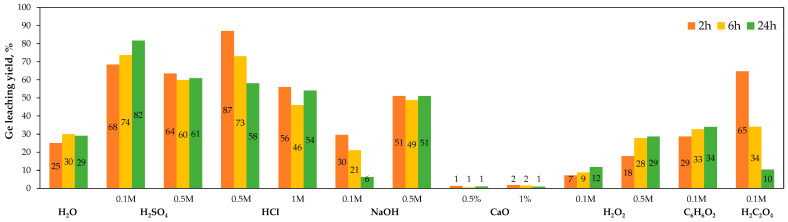
Effect of leachant type and concentration on germanium extraction rate (50 °C, L/S 5) from coal gasification fly ash (319 ppm Ge) at different leaching times, based on ref. [150].

Further studies [144,150,154] investigated germanium leaching under acidic (HCl, H_2_SO_4_), alkaline (NaOH, CaO, NaHCO_3_), and oxidizing (hydrogen peroxide H_2_O_2_) conditions, as well as with complexing agents (catechol C_6_H_6_O_2_, oxalic acid H_2_C_2_O_4_, tartaric acid H_2_C_2_O_4_). It was found that a highly efficient (75–90%) and fast (1–2 h) process with regular extraction yields for various ashes could be achieved with oxalic acid (0.16 M, 50 °C) as the leaching agent. Somewhat lower leaching rates (80–82%) were obtained with H_2_SO_4_, but these were accompanied by longer extraction durations and low selectivity. Other leaching agents showed low and/or irregular leaching yields for all tested parameters (concentration, time, type of fly ash). A representative comparison of time-dependent germanium extractions is shown in Figure 13.

##### From Laboratory to Pilot-Scale Implementation

**Figure 14:**
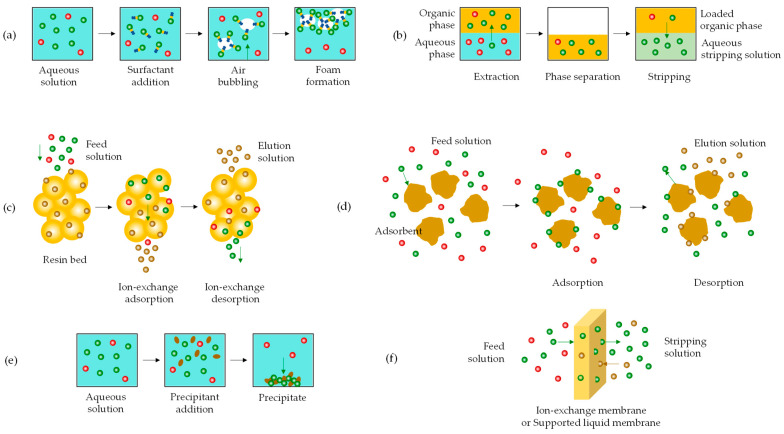
Schemes of hydrometallurgical unit processes for germanium recovery from leachates of coal gasification fly ash: (**a**) ion flotation, (**b**) solvent extraction, (**c**) ion-exchange, (**d**) adsorption, (**e**) precipitation, (**f**) membrane separation.

Germanium leaching can achieve high extraction efficiency, but it is non-selective and results in solutions with very low germanium ion concentrations. This creates challenges in recovering the target ion from the leachates and requires specific treatment methods based on specific germanium ion properties (Figure 14). Various separation methods were tested for the recovery of germanium from aqueous solutions containing catechol species to scale up the entire recovery process from the laboratory to the pilot-plant level. These involved ion flotation [152], solvent extraction [149,153,157], ion-exchange [158,159], adsorption [160], and precipitation [156,157].

Hernández-Expósito et al. [152] developed an ion flotation method using complexing agents (pyrogallol, catechol, resorcinol, hydroquinone) and dodecylamine surfactant as a collector for germanium recovery from aqueous solution (fly ash leached with water). It was found that the germanium separation was selective with respect to impurities (Ni, As, Sb), and the recoveries increased in the order: hydroquinone (20%) < resorcinol (34%) << catechol ~ pyrogallol (100%). Catechol (catechol/Ge concentration ratio of 3) was selected as the most efficient complexing agent (pH 4–7), ensuring highly selective and complete collection of germanium ions in the froth within 0.5 h.

**Figure 15:**
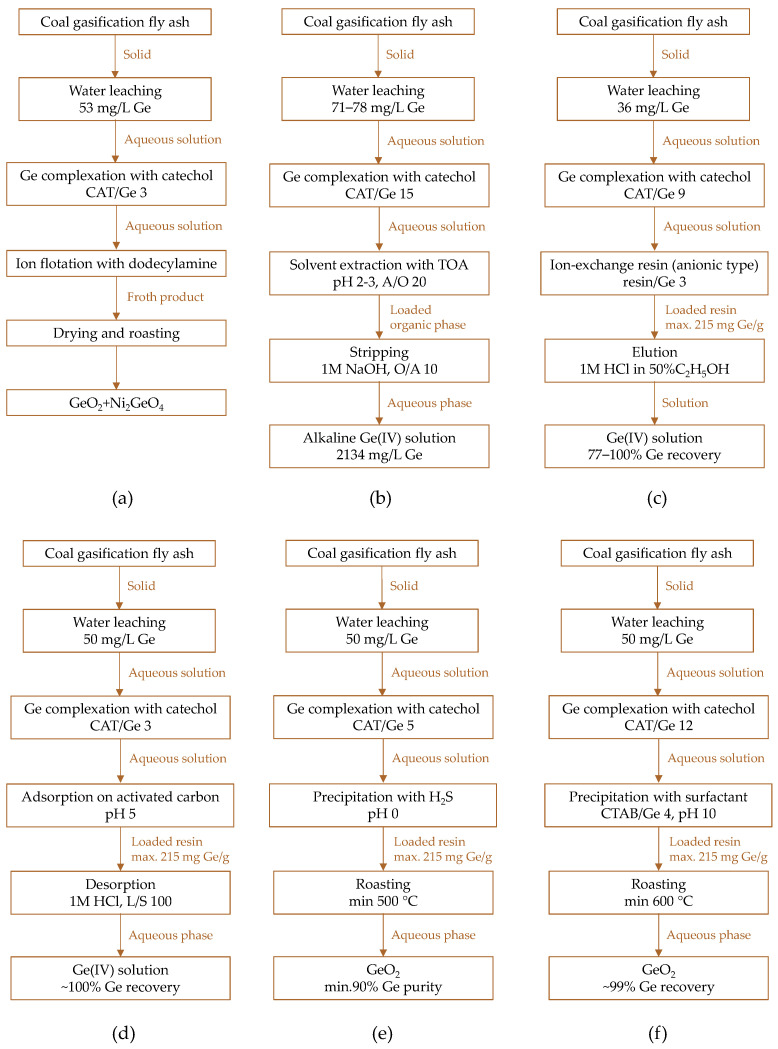
Schemes of hydrometallurgical germanium recovery from coal gasification fly ash (IGCC) and its water–catechol leachate using: (**a**) ion flotation, based on ref. [152], (**b**) solvent extraction, based on ref. [153], (**c**) ion-exchange, based on ref. [159], (**d**) adsorption, based on ref. [160], (**e**,**f**) precipitation, based on refs. [156,157].

The whole process followed the consecutive steps (Figure 15a):

(i) germanium leaching from the fly ash

hex-GeO_2_ + 2H_2_O → Ge(OH)_4_ (5)

(ii) complexation of germanium species with catechol

Ge(OH)_4_ + 3C_6_H_4_(OH)_2_ → [Ge(C_6_H_4_O_2_)_3_]^2−^ + 2H^+^ + 4H_2_O (6)

(iii) formation of froth product with dodecylamine

[Ge(C_6_H_4_O_2_)_3_]^2−^ + 2R-NH_2_ + 2H^+^ → (R-NH_3_)_2_[Ge(C_6_H_4_O_2_)_3_] (7)

The floated germanium complex was then dried and roasted at a minimum of 600 °C. This process resulted in a final product containing 53% GeO_2_ and 22% NiO, with crystalline phases of GeO_2_ and Ni_2_GeO_4_ being identified.

An alternative method for separating the anionic Ge(IV)–catechol chelate complex from water was proposed by Arroyo et al. [149,153,154]. They investigated solvent extraction with tri-n-octylamine (TOA, i.e., (C_8_H_17_)_3_N) in kerosene under various process conditions, including pH (2–3), catechol concentration (CAT/Ge 3–30), contact time (1–5 min), A/O volume ratios (2–4), TOA concentration (TOA/Ge 2–5), and the presence of impurity ions (As, Mo, Ni, Sb, V, Zn). They also studied the type and concentration of stripping agents (HCl, H_2_SO_4_, NaOH; 0.5–6 M) and A/O volume ratios (5–20). The best extraction results were achieved under slightly acidic conditions (pH 2–3) with a CAT/Ge ratio of 15 and a TOA/Ge ratio of 9, using an A/O volume ratio of 20. Under these conditions, 97% of germanium ions were extracted within 3 min:

2(C_8_H_17_)_3_N_org_ + [Ge(C_6_H_4_O_2_)_3_]^2−^_aq_ + 2H^+^_aq_ ↔ [(C_8_H_17_)_3_NH]_2_[Ge(C_6_H_4_O_2_)_3_]_org_ (8)

Stripping tests showed increasing efficiency in the following order of stripping agents: H_2_SO_4_ (43–63%) < HCl (72–80%) < NaOH (62–88%), with NaOH being the most effective stripping solution (Figure 15b):

[(C_8_H_17_)_3_NH]_2_[Ge(C_6_H_4_O_2_)_3_]_org_ + 2OH^−^_aq_ ↔ 2(C_8_H_17_)_3_N_org_ + [Ge(C_6_H_4_O_2_)_3_]^2−^_aq_ + 2H_2_O_aq_ (9)

**Figure 16:**
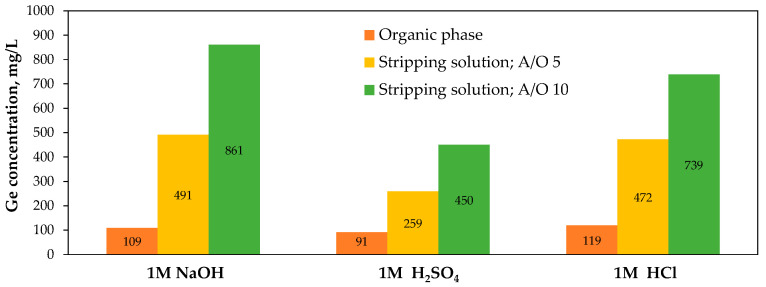
Germanium concentration in organic phase (tri-n-octylamine) and stripping aqueous solutions, based on ref. [154].

This process was further scaled up for pilot plant implementation [149,154], utilizing a leaching reactor (100 L), a complexation tank (30 L), and mixer-settler units for both extraction (15 L–2 L, settling area 120 cm^2^) and stripping (3 L–0.8 L, settling area 100 cm^2^). A 5 kg portion of IGCC fly ash was leached with water (L/S 5) at room temperature, achieving a 50% germanium extraction. The germanium-bearing leachate was then subjected to complexation with catechol (pH adjusted with 10% H_2_SO_4_), followed by solvent extraction using TOA (20 min, A/O 5), and stripping with NaOH (1 M, 30 min, A/O 5; three NaOH reuse tests). Under these conditions, 91–96% of germanium was recovered from the water leachate, with stripping yields reaching 75–97%. This resulted in the total recovery of germanium across three reutilizations of the stripping solution, with process efficiency gradually decreasing in successive reuse steps from 93% to 72%. Notably, the pilot-scale experiments also included the examination of stripping acids (Figure 16), with efficiency decreasing over three consecutive reuse tests from 58% to 29% for H_2_SO_4_ and from 56% to 34% for HCl.

The results [154] also revealed that, alongside germanium, several other metals, including As, K, Mg, Ni, Sb, Zn ions, were also extracted into the organic phase. However, their extraction was considerably lower than that of germanium (except for K ions), highlighting the selective nature of the process. Notably, Fe and Mg ions were either retained in the organic phase after stripping or precipitated as hydroxides in the NaOH medium [149]. The other elements either remained in the leachate after solvent extraction or were transferred into the stripping solutions, though generally at much lower concentrations than the germanium species [154].

The recovery of germanium using ion-exchange resins (Figure 15c) was also investigated as an alternative approach [158,159]. Adsorption tests were conducted on both synthetic solutions and real leachates containing the Ge(IV)–catechol complex, utilizing two quaternary ammonium macroporous resins, Amberlite IRA-900 and IRA-958 (both in chloride form, R_2_NR_3_Cl):

[Ge(C_6_H_4_O_2_)_3_]^2−^_aq_ + 2R_2_NR_3_Cl ↔ [R_2_NR_3_]_2_[Ge(C_6_H_4_O_2_)_3_] + 2Cl^−^_aq_ (10)

Conventional batch experiments combined with statistical techniques were used to assess the effects of pH, metal and catechol concentrations, and resin dosage on adsorption equilibria and to determine the optimal parameters. Germanium was selectively and efficiently extracted (~93%) by anionic resins when high catechol/Ge and resin/Ge ratios were applied (Figure 15c). Various eluting agents were tested, including aqueous solutions (HCl, NaOH, NaCl) and ethanol-based systems (HCl, H_2_SO_4_). Acid–ethanol solutions demonstrated significantly higher germanium recovery rates (71–94%) compared to aqueous solutions (22–63%). The most effective stripping solution for both anionic resins was 1 M HCl in 50% C_2_H_5_OH, with the additional benefit that chloride-containing solutions simultaneously regenerated the resins.

Marco-Lozar et al. [160] developed a new strategy for Ge–catechol adsorption on activated carbon as a simple, fast, and cost-effective method for germanium recovery from dilute solutions (Figure 15d). They investigated the adsorption process as a function of ionic strength, pH, contact time, type of activated carbon, and its activation with phosphoric acid or thermal treatment. The study found that decreasing the pH from 10 to 5 enhanced the adsorption process, but it was completely inhibited at pH 0 due to the destruction of the catechol complex at pH values below 3. This phenomenon was evident in the complete recovery of germanium from the adsorbent using an HCl solution. Reusing the same acid solution for the desorption step allowed germanium to be concentrated from an initial 50 mg/L to about 550 mg/L over 10–12 cycles. Notably, the adsorption process was selective for germanium, as the adsorption capacities for impurities such as As, Sb, and Si were very low.

In turn, Arroyo et al. [156,157] compared two methods for germanium precipitation from Ge–catechol complex-containing aqueous leachate (Figure 15e). They selected two precipitating agents:

(i) hydrogen sulfide, H_2_S, a conventional and inexpensive compound already generated as a by-product in an IGCC plant:

[Ge(C_6_H_4_O_2_)_3_]^2−^ + 2H_2_S + 2H^+^ ↔ GeS_2_↓ + 3C_6_H_4_(OH)_2_ (11)

(ii) cetyltrimethylammonium bromide CTAB, a surfactant that forms crystalline molecular complexes with various aromatic compounds, such as catechol:

[Ge(C_6_H_4_O_2_)_3_]^2−^ + 2CTA^+^ ↔ (CTA)_2_[Ge(C_6_H_4_O_2_)_3_]↓ (12)

It was found [156] that up to 100% of germanium could be precipitated from an aqueous solution acidified with 6 M HCl (pH 0) in a single sulfiding step (using H_2_S or a 1:1 H_2_S/CO mixture). However, increasing the solution pH or decreasing the H_2_S dosage (in the CO mixture) had detrimental effects. Surprisingly, the final product consisted of hexagonal GeO_2_ instead of the expected GeS_2_ or GeS, likely due to the high susceptibility of the sulfide to hydrolysis:

GeS_2_↓ + 2H_2_O → GeO_2_ + H_2_S (13)

A disadvantage of this method, however, is the co-precipitation of impurity species, mainly arsenic (100%) and antimony (30–100%), while nickel remained in the leachate. A final product of GeO_2_ with 91–93% purity was obtained (after roasting at 500–700 °C) if the leachates were pre-enriched by adsorption on activated carbon and/or solvent extraction.

An unconventional precipitation agent, the CTAB surfactant, was also investigated [156,157]. The Ge–catechol–CTA organic complex was able to bond almost 99% of germanium from an alkaline solution, while increasing the solution’s acidity reduced germanium recovery to only 40% at a pH of about 4. The organic complex was then roasted to produce GeO_2_ with up to 87% purity (Figure 15f). The purity of the final product could be improved if solvent extraction were incorporated into the process, with 90% GeO_2_ achieved from an HCl stripping solution. However, lower purity, 78% pure GeO_2_, was obtained from a NaOH stripping solution.

Water-based germanium leaching was revealed as an environmentally friendly method, but it lacked selectivity, leading to the extraction of impurities such as As, Sb, and Ni. Chimenos et al. [161] demonstrated that leaching in an oxidizing atmosphere (oxygen or air flow) reduces the presence of impurities in the leachate, resulting in a more selective germanium extraction. The concentration of germanium in the leachate was not significantly affected by the change between oxidizing and neutral (nitrogen) atmospheres. Under optimal conditions (90 °C, oxygen flow, at least 750 rpm), the leachates contained approximately 45 mg/L Ge, 30 mg/L Ni, 5 mg/L Sb, and no detectable As. The dissolved oxygen in the aqueous solution led to the formation of Fe–(oxy)hydroxide structures due to the oxidation of iron sulfides in the IGCC fly ash, which became the limiting step of the process. Notably, reducing the amount of dissolved oxygen in the aqueous slurry, as well as lowering both stirring speed and leaching temperature, diminished the oxidation of iron sulfides and the formation of Fe–(oxy)hydroxide structures, resulting in lower removal of metal and metalloid ions.

Recently, Fernández-Pereira et al. [144] conducted the valorization of a sustainable germanium extraction process (85% recovery) from IGCC fly ash using water as a leaching agent. The leached fly ash was subsequently used in the manufacture of fire-resistant boards containing 60% ash, thus avoiding its disposal in a landfill. The authors concluded that the real benefit of the developed process is not economic, but rather environmental, as approximately 300 g of Ge can be obtained from 1 ton of ash.

#### 5.3.2. Alternative Approach

**Figure 17:**
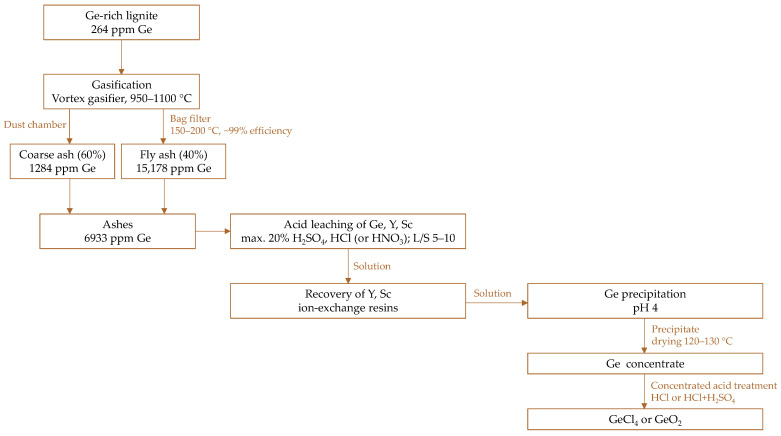
The flowsheet of germanium recovery from lignite gasification ashes, based on ref. [145].

Shpirt et al. [145] proposed a method for germanium recovery from ashes generated during the gasification of lignite. They conducted thermodynamic analysis (Figure 11) and experimental studies of the gasification process, which provided the basis for proposing the production of commercial products. The flowsheet of the complete process (Figure 17) involved gasification of germanium-rich lignite to yield coarse and fly ashes. Both ashes were collected together and subjected to acid leaching with moderately concentrated mineral acids to extract germanium, yttrium, and scandium. Rare metal ions were then selectively separated using ion-exchange resins (specific types were not shown), while germanium compounds were precipitated from the remaining solution by neutralizing to pH 4. The resulting precipitate, after drying, constituted a germanium concentrate that can be further processed using traditional methods with concentrated HCl or a HCl–H_2_SO_4_ mixture. Germanium can be recovered from the solutions as either GeCl_4_ or GeO_2_. The proposed process, though adapting already known techniques and equipment, was presented descriptively without providing detailed parameters or experimental results, which limits its real evaluation.

#### 5.3.3. Innovations

Selective separation of germanium from aqueous leachates of coal fly ash has been the subject of various methods utilizing ionic properties, such as solvent extraction [162], membrane processes [163], and dedicated adsorbents [164,165].

Haghighi et al. [162] investigated solvent extraction of germanium from simulated aqueous solutions containing tartaric acid, citric acid, oleic acid, or catechol as complexing agents. Three types of extractants were tested: Alamine 336 (tri-octyl/decyl amine), Aliquat 336 (N-methyl-N,N-dioctylchloride), and Cyanex 923 (phosphine oxide). In each extraction system, germanium was exclusively transferred from the aqueous to the organic phase, while other ions (Ni, Cd, Co, Zn) remained in the aqueous phase. Under comparable conditions, the germanium extraction efficiency for the aforementioned extractants followed the order: Aliquat 336 > Alamine 336 > Cyanex 923. Notably, nearly 100% germanium extraction was achieved with Aliquat 336 when tartaric and citric acids were used as complexing agents, while 85% extraction occurred in the presence of catechol. Oleic acid, however, almost completely inhibited germanium extraction. Various stripping agents were also tested, and among 0.1 M solutions of NH_4_Cl, NH_3aq_, NaOH, C_6_H_6_O_2_, C_6_H_8_O_7_, Na_2_SO_4_, and H_2_SO_4_, only sodium hydroxide yielded high stripping efficiency (nearly 92%).

This process was further developed [163] by using an extractant impregnated in supported liquid membranes. Innovative hollow fiber-supported liquid membrane exhibited an effective surface area 125,000 times greater than traditional flat-sheet supported liquid membranes, allowing for fast ion transport rates comparable to solvent extraction, while preventing phase dispersion. The effectiveness of PTFE and PVDF membranes containing Alamine 336 was compared with the hollow fiber membrane Liqui-Cel. In a traditional flat-sheet membrane setup, the feed phase (leachate) was separated from the strip phase (HCl), enabling the selective transport of target ions through ion exchange via the impregnated extractant. In the case of hollow fiber membrane, the organic carrier (Alamine 336) was dispersed in the HCl strip solution, forming a pseudoemulsion. This pseudoemulsion was circulated from the strip container to the lumen side of the membrane module, constantly supplying the organic phase into the membrane pores. The aqueous feed phase was circulated on the shell side. The feed phase volume was four times greater than the strip phase. It was found that the highest separation of germanium from impurity ions (Ni, Cd, Co, Zn) was achieved with membranes impregnated with 10 vol.% Alamine 336 and a 0.5 M HCl strip solution. The presence of tartaric acid (2.8 M) in the feed solution was a key factor enabling the transport of germanium due to the formation of anionic complexes. The faster transport of ions through the hollow fiber membrane compared to the flat-sheet membrane was confirmed.

Germanium-specific adsorbents were also synthesized and recently developed. Patel and Karamalidis [164] proposed a novel microwave-based synthesis method to functionalize polystyrene beads with catechol, nitrocatechol, and pyrogallol. The effectiveness of the adsorbents was then tested in aqueous solutions (20 mg/L Ge) with varying pH (1–10), demonstrating high selectivity for germanium recovery in slightly acidic solutions with pH values ranging from 1 to 3. The maximum adsorption capacities were observed at pH 3, in the following order (24 h): catechol-functionalized (30 mg/g) < pyrogallol-functionalized (37 mg/g) < nitrocatechol-functionalized (39 mg/g). Eight adsorption–desorption cycles were conducted, with the catechol-functionalized adsorbent (nearly 100%) showing consistent adsorption and desorption. However, the other two adsorbents exhibited a loss of capacity in the first cycle due to incomplete desorption. In subsequent cycles, both adsorption and desorption processes became consistent.

In turn, Wu et al. [165] synthesized a trihydroxy-functionalized titanium dioxide adsorbent, TiO_2_–NH_2_–3OH, using the Schiff base reaction method. The effectiveness of the adsorbent was tested in solutions with varying pH (3–13) and a germanium concentration of 20 mg/L, in the presence of ionic impurities (Zn, Co, Mn, Ni, Al). The adsorbent demonstrated selectivity and superior capture of germanium ions at pH 3, with a maximum germanium adsorption capacity of 73 mg/g. A high germanium desorption rate (nearly 81%) after five cycles was achieved by washing the loaded adsorbent with 0.5 M HCl.

## 6. Materials and Methods

The literature search was conducted between January and March 2025, focusing on keywords relevant to the research topic. This systematic review is based on 165 references, including peer-reviewed articles, books, and publicly available online information. Key databases, including Scopus, as well as specific publisher databases such as the American Chemical Society, IOP Science, MDPI, Royal Society of Chemistry, Taylor & Francis Online, Science Direct, Springer Link, and Wiley Online Library, were thoroughly examined. Additionally, freely accessible papers, statistical data, and institutional reports were included to obtain detailed information, such as germanium production, concentration in world coals, or current metal prices. Research articles, reviews, and books were selected based on an assessment of their titles, abstracts, and full texts. A comprehensive review of the relevant literature was also accomplished through the tree-research method, involving the analysis of citations and examination of supplementary materials (where available). Emphasis was placed on assessing the methodological approaches, main findings, and the applicability of studies to the current review framework, thereby establishing a basis for further analysis and discussion.

## 7. Conclusions

**Figure 18:**
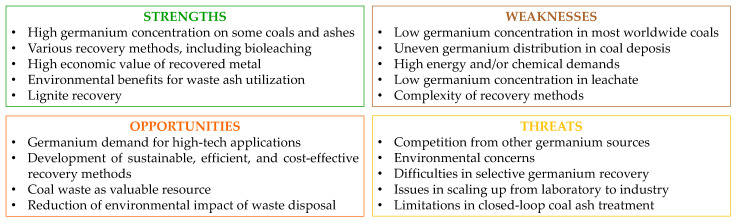
SWOT analysis of germanium hydrometallurgical recovery from coal by-products.

The potential for germanium recovery from coal, particularly from lignite and subbituminous coal, offers both promising opportunities and significant challenges (Figure 18). While germanium concentrations in global coal deposits are generally low (typically only a few ppm), its extraction could substantially increase the value derived from coal waste utilization.

Germanium-rich coal deposits are primarily found in China and Russia, where the metal is currently recovered from coal combustion fly ashes (which can contain several thousand ppm) using chlorination–distillation methods. This process requires specific conditions, as germanium, initially bound in organic matter, is transformed into a poorly soluble aluminosilicate phase during combustion.

Coal gasification fly ash also holds considerable potential, as germanium tends to accumulate in this by-product. Unlike coal combustion fly ash, this type of waste contains somewhat lower concentrations of germanium (typically several hundred ppm), but the germanium is present in easily leachable, water-soluble forms. Scaling the efficient and selective hydrometallurgical process remains a challenge, although promising results have been obtained in processes for recovering germanium bound in chelate complexes with catechol.

The development of innovative, selective recovery methods from leachates faces additional problems due to the low concentrations of germanium (typically in the tens of mg/L) and the presence of significant amounts of impurity metal ions, e.g., aluminum, iron, and zinc. While methods for producing high-purity germanium are still limited, GeO_2_ remains the most commonly proposed final product.

Despite global trends aimed at reducing coal use for energy production, coal remains an important resource, and coal ashes can represent a valuable source of germanium. The development of coal gasification technology has further highlighted the potential of this resource, as germanium can be easily leached from its fly ashes using water. A major challenge, however, is the development of selective and efficient methods for recovering germanium ions from highly diluted aqueous solutions. Future research should focus on specific selective methods based on ionic properties and using real leachates that contain various contaminating elements. These approaches could lead to more efficient recovery processes and contribute to the sustainable utilization of coal by-products.

## Figures and Tables

**Table 1:** Germanium concentrations in natural sources.

Category	Source	Concentration Range (Median)	Unit	Ref.
Space	Meteorites	0.01–2000	ppm	[28,29]
Earth	Core	(37)	ppm	[22]
	Bulk	(13.8)	ppm	[22,29]
	Continental crust	1.0–1.7 (1.4)	ppm	[29,30,31]
Environments	Soils *	0.02–379 (2.5)	ppm	[31,32,33]
	Rivers *	0.7–1487 (9–11)	ppt	[31,34]
	Lakes	0.06–6.8	ppt	[31]
	Hydrothermal waters	0.006–50	ppm	[22,31,35]
	Oceans	50–70 (60)	ppt	[29]
Ge-bearing minerals	Silicates	0.3–700	ppm	[29]
	Sulfides	0.05–5000	ppm	[22,29]
	Oxides, hydroxides	0.3–10,000	ppm	[29,36]
Plants	Field soil	0.001–2.8	ppm	[37]
	Greenhouse, soil amended with Ge	6.8–2086	ppm	[37]
	Aquatic environment *	7–220	ppm	[34]

* Including Ge contamination from WEEE recycling plant [32], non-ferrous metallurgy [34], or coal combustion [31,34].

**Table 2:** Germanium concentration in coals worldwide.

Continent	Country	Coal Type	Concentration (Mean), ppm	Refs.
World	–	hard (bituminous, anthracite)	(2.4 ± 0.2)	[54]
		brown (subbituminous, lignite)	(2.0 ± 0.1)	[54]
Africa	Bostwana	no data	0.9–9	[55]
	Nigeria	bituminous, subbituminous	0.2–5	[55]
	Tanzania	subbituminous or no data	1–10	[55]
	South Africa	no data	1–16	[56]
Antarctica	-	bituminous–subbituminous	1–81 (11)	[55]
Asia	Afghanistan	bituminous, lignite	0.2–11	[55]
	China	bituminous, subbituminous, lignite	1–2500 (2.7)	[46]
	India	bituminous, lignite	2–343	[55,71]
	Mongolia	bituminous, subbituminous, lignite	0.1–1.5	[55]
	Vietnam	anthracite	0.3–1	[55]
Australia & Oceania	Australia	bituminous, lignite	0.5–58	[55,63]
	New Zealand	bituminous, subbituminous, lignite	0.1–3	[55]
Europe	Czech Republic	bituminous, subbituminous, lignite	0.1–631 (27)	[55]
	Hungary	bituminous, subbituminous, lignite	0.3–5	[55]
	Poland	bituminous	0.2–2	[61,62]
	Turkey	bituminous, subbituminous, lignite	0.2–23 (3.7)	[55]
	United Kingdom	bituminous	1–48 (8)	[55,72,73]
North America	Canada	no data	0.1–2	[55]
	United States	bituminous, subbituminous, lignite	0.03–235 (5.5/7.2)	[49,70]
South America	Argentina	bituminous	0.7–6	[55]
	Brazil	bituminous, subbituminous	1–99 (13)	[55]
	Colombia	bituminous, subbituminous	0.3–7	[55]
	Peru	anthracite	0.1–2	[55]
	Venezuela	no data	0.1–20 (3)	[55]

**Table 3:** Germanium concentration (ppm) in coal gangue (Huanglong Coalfield, China) [101].

Mine	Sampling Location in Coal Seam		
	Roof	Parting	Floor
Bindong	10–35	4–28	8–38
Guojiahe	10–45	1–33	8–38
Yuanzigou	1–33	1–33	2–35
Zhaoxian	5–31	4–30	8–33

**Table 4:** Germanium concentration (ppm) in coals and combustion ashes.

Region	Coal	Coal Ash	Fly Ash	Bottom Ash (Slag)	Refs.
Brazil, power plants	1.0–6.4	–	1.6–41.8	1.1–7.2	[108]
Bulgaria, Pernik (feed coal)	<2.0	<3.7	–	–	[109]
Bulgaria, Pernik (high-grade coal)	<4.5	<24	–	–	[109]
China, Chongqing power plant	–	–	4.5	1.3 (1.4)	[110]
China, Lincang	1294/825	3902/7100	38,964	(96.3)	[57,111]
China, Mangniuhai Coalfield	107 ± 45	–	302 ± 58	184 ± 74	[105]
China, Wulantuga	273/727	2820/1643	–	–	[57,111]
Greece, Kardia	–	154–186 ^L^	1.4 ^P^	–	[112,113]
Greece, Megalopolis	–	134–185 ^L^	3.3 ^P^	–	[112,113]
India, Jharkhand power plant	–	7.2 ^L^/18.6	–	–	[114]
Italy, power plants	–	–	7–18	–	[115]
Mexico, Coahuila power plant	–	–	12	–	[116]
Netherlands, power plants	–	–	4–35	–	[115]
Poland, Lublin Coal Basin	–	50–1720	–	–	[117]
Russia, Kuznetsk Basin	0.9	7.5	–	–	[118]
Russia, Minusinsk Basin	1.5/1.2	14.3/11.4	–	–	[119]
Russia, Spetsugli	1025	4906	1120/24,600	(270)	[57]
Russia, Western Siberia	10–67/39–198 ^L^	62–263/2389–6500 ^L^	–	–	[68]
Spain, power plants	1.5	–	1–132	(4.0)	[120,121,122]
Spain, Puertollano Basin	1–56	61	–	–	[123]
South Africa	56.5/122	1135/1330	7.5–111 ^P^	–	[56]
Turkey, Cayirhan power plant	3.3 ± 1.3	6.6 ± 2.6	6.2 ± 1.9	2.4 ± 0.3	[124]
Turkey, Kangal power plant	0.1–1.0	–	0.4–2.0	0.2–2.6	[125]
Turkey, Seyitömer power plant	0.01–1.4	–	1.2–3.6	0.01–1.2	[126]
Turkey, Tunçbilek power plant	0.7–2.4	–	1.3–4.9	0.9–1.8	[126]
United Kingdom	51–70	230–1500	40–14,000 ^P^	–	[48]
USA, Colorado	–	5–400	–	–	[127]
USA, Kentucky (Gray Hawk)	6.97	175	–	–	[79]
USA, Montana	–	5–47,000	–	–	[127]
USA, North Dakota	–	5–430	–	–	[127]
USA, Ohio	–	5–2600	–	–	[127]
USA, Wyoming	–	5–650	–	–	[127]

^L^—Lignite. ^P^—Power plants.

**Table 5:** Industrial germanium recovery from coal, based on [46,57].

Process Details	Plant Localization		
	Wulantuga, China	Lincang, China	Spetsugli, Russia
Designed capacity	100 t/y	39–48 t/y	21 t/y
Coal combustion	vortex furnace	chain conveyor furnace	flare-layered boiler
Combustion intensity	156 kg/m^2^	no data	no data
Boiler temperature	1200 °C	no data	no data
Fly ash collection	baghouse filter	baghouse fabric filter, 110–120 °C, efficiency 99.8%	no data
Ge concentration in fly ash	14,870 ppm	38,964 ppm	24,600 ppm
Ge concentration on an ash basis	35,170 ppm	46,580 ppm	29,078 ppm
Products	different, including high-purity zone-refined germanium ingots (99.99999–99.999999% Ge)	no data	no data

**Table 6:** Germanium extraction and recovery from coal fly ash.

Extraction Method	Leaching Rate	Recovery Method	Recovery Rate	Ref.
Acid leaching	no data	Solvent extraction (dihydroxamic acid in sulfonated kerosene)	99%	[137] *
H_2_SO_4_ leaching	no data	Salt pyrolysis to produce GeO_2_	no data	[137] *
H_2_SO_4_ + acid (or salt) leaching	85–90%	no data	no data	[137] *
Two-step acid leaching: (1) dilute for Ge, (2) concentrated for Al	no data	High-temperature decomposition of double salt	no data	[137] *
Ash chlorination with NH_4_Cl, HCl leaching	no data	Solvent extraction (dihydroxamic acid)	min. 95%	[137] *
Ash suspended in 8–9.6 M HCl in a foam column (air bubbling), room temperature	no data	Absorption of evaporated GaCl_4_ in a foam column with alkali solution or nonpolar solvent (e.g., CCl_4_, gasoline). HCl addition followed by GeCl_4_ distillation.	no data	[93]
Ash reductive smelting (to 3–4% Ge alloy), leaching in FeCl_3_ + Cl_2_	no data	Addition of H_2_SO_4_ (to 14 M) to chloride Ge-bearing solution, GeCl_4_ distillation	no data	[93]

* Original paper not available.

**Table 7:** Germanium concentration (ppm) in raw coal and coal gasification solid products.

Region/Source	Coal Type	Coal	Fly Ash	Slag (Bottom Ash)	Refs.
Poland/Laboratory	bituminous	0.2–0.3	0.8–1.0	0.3–0.4	[147]
Poland/Laboratory	lignite	0.2	0.8	0.5	[147]
Russia/Laboratory	lignite	263	15,178/1284 *	317	[145]
Spain/IGCC plant	bituminous + petcoke	–	194–420	–	[148,149]
Spain/IGCC plant	bituminous (+petcoke)	10.7–21	174–356	–	[150]

* Highly dispersed ash/Coarse ash.

## Data Availability

No new data were created.
